# Supplementary material for: A Systematic Review of the Efficacy and Safety of Direct Oral Anticoagulants in Atrial Fibrillation Patients with Diabetes Using a Risk Index
Source: J Clin Med. 2021 Jun 29;10(13):2924. doi: 10.3390/jcm10132924 (PMC8268134; doi:10.3390/jcm10132924)
Supplement: Supplementary file 1 [file jcm-10-02924-s001.zip › jcm-1262759-supplementary.pdf]

Efficacy and safety of direct oral anticoagulants in patients with atrial fibrillation and diabetes using a risk index.
A Systematic Review.

Running title: Risk index of efficacy and safety of oral anticoagulants in diabetic patients with atrial fibrillation.

Domenico Acanfora, MD, Marco Matteo Ciccone, MD, Valentina Carlomagno, MD, Pietro Scicchitano, MD, Chiara Acanfora, MD, Massimo Uguccioni MD, Gerardo Casucci, MD.

Supplementary data

Details of the search strategy

Table S1: Search strategy according to PRISMA-P

**OVERVIEW**	
Interface	Ovid
Databases	EBM Reviews - Cochrane Central Register of Controlled Trials<br>Ovid Embase<br>Ovid MEDLINE<br><br>Ovid MEDLINE Daily<br>Ovid MEDLINE In-Process & Other Non-Indexed Citations<br><br>*Note: Duplicates between databases were removed in Ovid.*<br>PubMed
Date of search	February 1th 2020
Alerts	Monthly search updates began February 1th 2020 and ran until project completion.
Study types	Randomized Controlled Trials
Limits	English language<br>Publication years 2002-present

	Conference abstracts omitted

Table S2: Medline

Ovid MEDLINE(R) In-Process & Other Non-Indexed Citations, Ovid MEDLINE(R) Daily, Ovid MEDLINE(R) and Ovid OLDMEDLINE(R) 2002 to Present.

ID	PICOS	Category	Search terms
#1	P	Indication (VTEx)	venous thromboembolism/
#2			deep vein thrombosis/
#3			((vein or venous) adj thromb$).ti,ab.
#4			((pulmonary or lung) adj embol$).ti,ab.
#5			(dvt or vte or dvts or vtes).ti,ab.
#6			or/1-5
#7		Indication (SPAF)	(spaf or nvaf).ti,ab.
#8			Atrial Fibrillation/ or Atrial Flutter/
#9			((Atrial or atrium or auricular) adj2 fibrillat$).ti,ab.
#10			(AF or A-fib or a fib).mp.
#11			or/7-10
#12		Indication (VTEp OS)	exp arthroplasty, replacement, knee/
#13			(knee adj (replacement or arthroplasty or prosthesis or joint)).mp.
#14			TKR.mp.
#15			exp arthroplasty, replacement, hip/
#16			(hip adj (replacement or arthroplasty or prosthesis or fracture)).mp.
#17			THR.mp.
#18			or/12-17
#19	Hits of P	6 or 11 or 18	270,892
#20	I and C	Interventions and Comparators	antithrombins/
#21			noac$.mp.
#22			new oral anticoagulant$.mp.
#23			doac$.mp.
#24			direct oral anticoagulant$.mp.
#25			New orally active anticoagulant$.mp.
#26			Novel oral anticoagulant$.mp.
#27			21 or 22 or 23 or 24 or 25 or 26
#28			IIa inhibitor$.mp.
#29			direct thrombin inhibitor$.mp.
#30			28 or 29
#31			Factor Xa Inhibitor/
#32			Xa inhibitor$.mp.
#33			fxa inhibitor$.mp.
#34			factor 10a inhibitor$.mp.
#35			31 or 32 or 33 or 34
#36			(dabigatran or BIBR$1048 or pradax* or prazax*).mp.
#37			(edoxaban or DU$176b or lixiana).mp.
#38			(apixaban or BMS$562247 or eliquis).mp.
#39			(rivaroxaban or BAY 59$7939 or xarelto).mp.
#40			36 or 37 or 38 or 39
#41	Hits of I and C	20 or 27 or 30 or 35 or 40	
#42	S	Observational studies	Epidemiologic studies/
#43			exp case control studies/
#44			exp cohort studies/
#45			Case control.tw.
#46			(cohort adj (study or studies)).tw.
#47			Cohort analy$.tw.
#48			(Follow up adj (study or studies)).tw.
#49			(observational adj (study or studies)).tw.
#50			Longitudinal.tw.

ID	PICOS	Category	Search terms
#51			Retrospective.tw.
#52			Cross sectional.tw.
#53			Cross-sectional studies/
#54			(cohort$1 or cross section$ or crosssection$ or (real adj1 (world or life))).tw.
#55			(claim$ adj1 (data or analys*)).tw.
#56			Registries/
#57			(database$ or registry or registries or effectiveness or prospective stud$).tw.
#58			or/42-57
#59			Randomized controlled trial/ or Randomization/
#60			Comment.pt.
#61			Letter.pt.
#62			Editorial.pt.
#63			Case reports.pt.
#64			59 or 60 or 61 or 62 or 63
#65	Hits of S	58 not 64	
#66	Hits of P and I and S	19 and 41 and 65	
#67	Limits	Humans	animals/
#68		66 not 67	
#75			remove duplicates from 68

Table S3: Embase

Embase Classic and Embase 1947 to 2015 Week 21 using Ovid plateform

ID	PICOS	Category	Search terms
#1	P	Indication (VTEx)	venous thromboembolism/
#2			deep vein thrombosis/
#3			((vein or venous) adj thromb$).ti,ab.
#4			((pulmonary or lung) adj embol$).ti,ab.
#5			(dvt or vte or dvts or vtes).ti,ab.
#6			or/1-5
#7		Indication (SPAF)	(spaf or nvaf).ti,ab.
#8			Atrial Fibrillation/ or Atrial Flutter/
#9			((Atrial or atrium or auricular) adj2 fibrillat$).ti,ab.
#10			(AF or A-fib or a fib).mp.
#11			or/7-10
#12		Indication (VTEp OS)	exp arthroplasty, replacement, knee/
#13			(knee adj (replacement or arthroplasty or prosthesis or joint)).mp.
#14			TKR.mp.
#15			exp arthroplasty, replacement, hip/
#16			(hip adj (replacement or arthroplasty or prosthesis or fracture)).mp.
#17			THR.mp.
#18			or/12-17
#19	Hits of P	6 or 11 or 18	
#20	I and C	Interventions and Comparators	antithrombins/
#21			noac$.mp.
#22			new oral anticoagulant$.mp.
#23			doac$.mp.
#24			direct oral anticoagulant$.mp.
#25			New orally active anticoagulant$.mp.
#26			Novel oral anticoagulant$.mp.
#27			21 or 22 or 23 or 24 or 25 or 26
#28			IIa inhibitor$.mp.

ID	PICOS	Category	Search terms
#29			direct thrombin inhibitor$.mp.
#30			28 or 29
#31			Factor Xa Inhibitor/
#32			Xa inhibitor$.mp.
#33			fxa inhibitor$.mp.
#34			factor 10a inhibitor$.mp.
#35			31 or 32 or 33 or 34
#36			(dabigatran or BIBR$1048 or pradax* or prazax*).mp.
#37			(edoxaban or DU$176b or lixiana).mp.
#38			(apixaban or BMS$562247 or eliquis).mp.
#39			(rivaroxaban or BAY 59$7939 or xarelto).mp.
#40			36 or 37 or 38 or 39
#41	Hits of I and C	20 or 27 or 30 or 35 or 40	
#42	S	Observational studies	Epidemiologic studies/
#43			exp case control studies/
#44			exp cohort studies/
#45			Case control.tw.
#46			(cohort adj (study or studies)).tw.
#47			Cohort analy$.tw.
#48			(Follow up adj (study or studies)).tw.
#49			(observational adj (study or studies)).tw.
#50			Longitudinal.tw.
#51			Retrospective.tw.
#52			Cross sectional.tw.
#53			Cross-sectional studies/
#54			(cohort$1 or cross section$ or crosssection$ or (real adj1 (world or life))).tw.
#55			(claim$ adj1 (data or analys*)).tw.
#56			Registries/
#57			(database$ or registry or registries or effectiveness or prospective stud$).tw.
#58			or/42-57
#59			Randomized controlled trial/ or Randomization/
#60			Comment.pt.
#61			Letter.pt.
#62			Editorial.pt.
#63			Case reports.pt.
#64			59 or 60 or 61 or 62 or 63
#65	Hits of S	58 not 64	
#66	Hits of P and I and S	19 and 41 and 65	
#67	Limits	Humans	animals/
#68		66 not 67	
#75			remove duplicates from 68

Table S4:

Table S4 (a): Cochrane

Cochrane Database of Systematic Reviews, Database of Abstracts of Reviews of Effect, Cochrane Central Register of Controlled Trials, Health Technology Assessment Database and NHS Economic Evaluation Database

ID	PICOS	Category	Search terms
#1	P	Indication (VTEx)	[mh "Venous Thromboembolism"]
#2			[mh "deep vein thrombosis"]
#3			((vein or venous) near thromb*)
#4			((pulmonary or lung) near embol*)
#5			(dvt or vte or dvts or vtes)
#6			#1 or #2 or #3 or #4 or #5
#7		Indication (SPAF)	(spaf or nvaf):ti,ab
#8			[mh "Atrial Fibrillation"]
#9			[mh "Atrial Flutter"]
#10			((Atrial or atrium or auricular) near/2 fibrillat*)
#11			(AF or A-fib or a fib)
#12			#7 or #8 or #9 or #10 or #11
#13		Indication (VTEp OS)	[mh "Arthroplasty, Replacement, Knee"]
#14			(knee near (replacement or arthroplasty or prosthesis or joint))
#15			(TKR)
#16			[mh "Arthroplasty, Replacement, Hip"]
#17			(hip near (replacement or arthroplasty or prosthesis or fracture))
#18			(THR)
#19			#13 or #14 or #15 or #16 or #17 or #18
#20	Hits of P	#6 or #12 or #19	
#21	I and C	Interventions and Comparators	[mh antithrombins]
#22			(noac*)
#23			(new oral anticoagulant*)
#24			(doac*)
#25			(direct oral anticoagulant*)
#26			(New orally active anticoagulant*)
#27			(Novel oral anticoagulant*)
#28			#22 or #23 or #24 or #25 or #26 or #27
#29			(IIa inhibitor*)
#30			(direct thrombin inhibitor*)
#31			#29 or #30
#32			[mh "Factor Xa Inhibitors"]
#33			(Xa inhibitor*)
#34			(fxa inhibitor*)
#35			(factor 10a inhibitor*)
#36			#32 or #33 or #34 or #35
#37			(dabigatran or BIBR$1048 or pradax* or prazax*)
#38			(edoxaban or DU$176b or lixiana)
#39			(apixaban or BMS$562247 or eliquis)
#40			(rivaroxaban or BAY 59$7939 or xarelto)
#41			#37 or #38 or #39 or #40
#42	Hits of I and C	#21 or #28 or #31 or #36 or #41	
#43	S	Observational studies	[mh "Epidemiologic studies"]
#44			[mh "case control studies"]
#45			[mh "cohort studies"]
#46			(Case control)
#47			(cohort near (study or studies))
#48			(Cohort analy*)
#49			(Follow up near (study or studies))
#50			(observational near (study or studies))
#51			(Longitudinal)
#52			(Retrospective)
#53			(Cross sectional)
#54			[mh "Cross-sectional studies"]
#55			(cohort* or cross section* or crosssection* or (real near/1 (world or life)))
#56			(claim* near/1 (data or analys*))
#57			[mh Registries]

ID	PICOS	Category	Search terms
#58			(database* or registry or registries or effectiveness or prospective stud*)
#59			#43 or #44 or #45 or #46 or #47 or #48 or #49 or #50 or #51 or #52 or #53 or #54 or #55 or #56 or #57 or #58
#60			[mh "Randomized controlled trial"]
#61			[mh Randomization]
#62			(Comment):pt
#63			(Letter):pt
#64			(Editorial):pt
#65			(Case reports):pt
#66			#60 or #61 or #62 or #63 or #64 or #65
#67	Hits of S	#59 not #66	
#68	Hits of P and I and S	#20 and #42 and #67	
#69	Limits	Humans	[mh Animals]
#70		#68 not #69	

Table S4 (b): Trial Registers

Trial Registers (date: 01-09-2018)	
Source	*Search Strategy*
https://www.clinicaltrialsregister.eu/	(Dabigatran OR Pradax OR Pradaxa OR apixaban OR Eliquis OR rivaroxaban OR Xarelto) AND (warfarin OR Coumadin OR acenocoumarol OR Sintrom OR Sinthrome) AND Atrial Fibrillation
https://clinicaltrials.gov/	(Dabigatran OR Pradax OR Pradaxa OR apixaban OR Eliquis OR rivaroxaban OR Xarelto) AND (warfarin OR Coumadin OR acenocoumarol OR Sintrom OR Sinthrome) AND Atrial Fibrillation

Table S4 (c): Grey Literature

Grey Literature (date: 01-09-2018)	
Source	*Search Strategy*

http://www.opengrey.eu/search/	(Dabigatran OR Pradax OR Pradaxa OR apixaban OR EliquisOR rivaroxaban OR Xarelto) AND (warfarin OR Coumadin ORacenocoumarol OR Sintrom OR Sinthrome) AND Atrial Fibrillation
http://www.greylit.org/	(Dabigatran OR Pradax OR Pradaxa OR apixaban OR EliquisOR rivaroxaban OR Xarelto) AND (warfarin OR Coumadin ORacenocoumarol OR Sintrom OR Sinthrome) AND Atrial Fibrillation

Table S5:

Table **S5 (a): Risk of Bias Assessment**

Publication: Dabigatran versus Warfarin in Patients with Atrial Fibrillation. N Engl J Med2009;361:1139-51.<br>*Authors:* Connolly SJ, Ezekowitz MD, Wallentin L.				
**Domain**	**Support for Judgement**	**Review Author's Judgement**		
**Selection Bias**				
Random Sequence Generation	Insufficient information in article or rationale and design publication	High Risk	Unclear	Low Risk
Allocation Concealment	Central interactive automated telephone system	High Risk	Unclear	Low Risk
**Performance Bias**				
Blindings of partecipants	Incomplete blinding:<br>- Blinded to Dabigatran dose<br>- Unblinded with respect to Dabigatran or warfarin assignment	High Risk	Unclear	Low Risk

And personnel	All investigators, members of coordinating center, the operation committee, the stering committee, the event adjudication committee, and the sponsor remain blinded to treatment level analyses of efficacy and safety. Only the data and safety monitoring board (DSMB) and the DSMB-statistician have access to the randomization code and by-treatment event rates			
**Detection Bias**				
Blinding of Outcome assestment	Adjudication of endpoints is blinded to drug assignment; by a blinded adjudication committee.	High Risk	Unclear	Low Risk
**Attrition Bias**				
Incomplete Outcome data	All analyses based on intention-to-treatprinciple. 20 patients (out of 18.113) lost tofollow-up.	High Risk	Unclear	Low Risk
**Incomplete Bias**				
Selective reporting	Study protocol available and all pre-specified outcomes are reported.<br>The unexpectedly different rates of myocardial infarction and gastrointestinal bleeding among the three treatment groups support an absence of bias.<br>To detect possible unreported events, symptom questionnaires were regularly administered to patients, and adverse-event and hospitalization reports were	High Risk	Unclear	Low Risk

	scrutinized for unreported primary or secondary outcomes			

Table **S5 (b): Risk of Bias Assessment**

Publication: Rivaroxaban versus Warfarin in Nonvalvular Atrial Fibrillation. N Engl J Med2011;365:883-91.*Authors:* Patel MR, Mahaffey KW, Califf RM.				
**Domain**	**Support for Judgement**	**Review Author's Judgement**		
**Selection Bias**				
Random Sequence Generation	Insufficient information in article or rationale and design publication.	High Risk	Unclear	Low Risk
Allocation Concealment	Central 24-hour, computerized, automated voice-response system.	High Risk	Unclear	Low Risk
**Performance Bias**				
Blindings of partecipants And personnel	Double-blind, double-dummy. A point-of-care device was used to either generate real INR values or sham values. Sham INR results were generated by means of a validated algorithm reflecting the distribution of values in warfarin-treated patients with characteristics similar to those in the study population.	High Risk	Unclear	Low Risk
**Detection Bias**				
Blinding of Outcome assestment	An independent clinical end-point committee applied protocol definitions to adjudicate all suspected cases of stroke, systemic embolism, myocardial infarction, death and bleeding	High Risk	Unclear	Low Risk

	events that contributed to the prespecified end points.			
**Attrition Bias**				
Incomplete Outcome data	The primary efficacy analysis (noninferiority) will be undertaken in the per-protocol population, which comprises all randomized patients who have received study drug, except those who have major protocol violations before a primary end point event. The analysis population consists of randomized subjects who have taken at least one dose of study drug. Testing for noninferiority and superiority was also performed in the intention-to-treat population, which included all patients who underwent randomization and were followed for events during treatment or after premature discontinuation 32 patients lost to follow up (18 in rivaroxaban group, 14 in warfarin group). The proportions of patients who permanently stopped their assigned therapy before an end-point event and before the termination date were 23.7% in the rivaroxaban group and 22.2% in the warfarin group. Because of violations in GCP guidelines at one site that made the data unreliable, 93 patients (50 in the rivaroxaban group and 43 in the warfarin group) were excluded from all efficacy analyses before unblinding.	High Risk	Unclear	Low Risk
**Incomplete Bias**				

Selective reporting	Study protocol available and all pre-specified outcomes are reported. When randomized, all patients will be observed for the duration of the study to ascertain clinical events. Patients will be seen at 1, 2, and 4 weeks, and every month thereafter for detection of primary efficacy end point events, as well as TIA, MI, bleeding complications, procedures, and vital status evaluation. A standardized questionnaire and examination will be used to screen for stroke symptoms and clinical events that will prompt further evaluation.	High Risk	Unclear	Low Risk

Table **S5 (d): Risk of Bias Assessment**

Publication: Apixaban versus Warfarin in Patients with Atrial Fibrillation. N Engl J Med 2011;365:981-92.*Authors:* Granger CB, Alexander JH, Wallentin L.				
**Domain**	**Support for Judgement**	**Review Author's Judgement**		
**Selection Bias**				
Random Sequence Generation	Insufficient information in article or rationale and design publication.	High Risk	Unclear	Low Risk
Allocation Concealment	Insufficient information in article or rationale and and design publication.	High Risk	Unclear	Low Risk
**Performance Bias**				

Blindings of partecipants And personnel	Double-blind, double-dummy. Blinded treatment is maintained through the use of sham international normalized ratios in patients receiving apixaban.	High Risk	Unclear	Low Risk
**Detection Bias**				
Blinding of Outcome assestment	An independent, blinded, clinical events committee (CEC)adjudicates all suspected hemorrhagic and non- hemorrhagicstrokes, TIAs, systemic emboli, major and clinically relevantnon-major bleeding, myocardial infarction, and cause ofdeath. Using prespecified event definitions and agreed uponevent adjudication criteria, the CEC adjudicates suspectedevents based on the preponderance of the evidence and theclinical knowledge and experience of the physicianreviewers.	High Risk	Unclear	Low Risk
**Attrition Bias**				
Incomplete Outcome data	The primary and secondary efficacy analyses included all patients who underwent randomization (intention-to-treat population) and included all events from the time of randomization until the cutoff date for efficacy outcomes. The analyses of bleeding events included all patients who received at least one dose of a study drug and included all events from the time the first dose	High Risk	Unclear	Low Risk

	of a study drug was received until 2 days after the last dose was received. In a modified intention-to-treat sensitivity analysis, we analyzed bleed- ing events that occurred in patientswho received at least one dose of a study drug andincluded all events from the time of randomizationuntil January 30, 2011.Data on vital status at the end of the trial were missing for 380 patients (2.1%). The absence of data on vital statuswas due to withdrawal of consent in the case of 92 patientsin the apixaban group (1.0%) and 107 patients in thewarfarin group (1.2%) and was due to loss to follow-up inthe case of 35 patients in the apixaban group (0.4%) and34 in the warfarin group (0.4%)			
**Incomplete Bias**				
Selective reporting	Study protocol available and all pre-specifiedoutcomes are reported.<br><br>Visits every 3 months included an assessment of clinicaloutcomes and adverse events. For each patient who waslost to follow-up or who withdrew consent, attempts weremade to determine vital status at the end of the trial.	High Risk	Unclear	Low Risk

Table **S5 (e): Risk of Bias Assessment**

Publication: Edoxaban versus Warfarin in Patients with Atrial Fibrillation. *Authors:* Giugliano RP, Ruff CT, Braunwald EMPH				
**Domain**	**Support for Judgement**	**Review Author's Judgement**		
**Selection Bias**				
Random Sequence Generation	Insufficient information in article or rationale and designpublication.	High Risk	Unclear	Low Risk
Allocation Concealment	All subjects are randomized through an interactive voice/Webresponse system.	High Risk	Unclear	Low Risk
**Performance Bias**				
Blindings of partecipants And personnel	Double-blind, double-dummy.<br><br>Blinded treatment is maintained through the use of shaminternational normalized ratios in patients receiving edoxaban.	High Risk	Unclear	Low Risk
**Detection Bias**				
Blinding of Outcome assestment	Cause of death, stroke, SEE, MI, bleeding, and hepatic events areadjudicated by members of an independent Clinical EventsCommittee blinded to treatment allocation.	High Risk	Unclear	Low Risk
**Attrition Bias**				
Incomplete Outcome	The primary analysis tests that ≥1edoxaban treatmentexposure is noninferior to warfarin	High Risk	Unclear	Low Risk

data	using a pairwisecomparison (modified ITT).<br><br>Further sensitivity analyses are performed using the ITT cohortincluding all events occurring while in study and the on-treatment analytic approach based on the "per protocol principle".<br><br>Complete information on the primary end point was ascertained for99.5% of the total 56,346 patient-years of potential follow-up.Rate of missing data (0.5%).<br><br>One patient was lost to follow-up, and 244 patients withdrew consentto follow-up; 182 of these patients had no known primary-end-pointevent and were not known to be dead.			
**Incomplete Bias**				
Selective<br><br>*reporting*	protocol available and all pre-specified outcomes are reported.<br><br>During follow-up visits, subjects are assessed for adverseevents, study end points, INR measured in a blinded fashion,and periodic safety laboratory tests (creatinine, liver function) sent to the central laboratory.	High<br><br>Risk	Unclear	Low Risk

Table **S6: Data Extraction Form**

**NAME INVESTIGATOR FOR DATA EXTRACTION:**	
**STUDY ID**	
Study nr<br><br>*Name of study*<br><br>*Publication year*<br><br>*Reference*	
**METHODOLOGY**	
FU duration(y)<br><br>*Number of patients*	
**BASELINE CHARACTERISTICS**	
Diabetics (n); (%)	
**OUTCOMES**	
Stroke/Syst Embolism (SEE) (n)<br>*Cardiovascular Death (n)*<br>*Major bleeding (n)*<br>*ISTH*<br><br>*ISTH modified*	
